# Differential health impact of intervention programs for time-varying disease risk: a measles vaccination modeling study

**DOI:** 10.1186/s12916-022-02242-2

**Published:** 2022-03-09

**Authors:** Allison Portnoy, Yuli Lily Hsieh, Kaja Abbas, Petra Klepac, Heather Santos, Logan Brenzel, Mark Jit, Matthew Ferrari

**Affiliations:** 1 Center for Health Decision Science, Harvard T.H. Chan School of Public Health, 718 Huntington Avenue 2nd Floor, Boston, MA 02115 USA; 2 Interfaculty Initiative in Health Policy, Harvard University, Cambridge, 02138 USA; 3 Department of Infectious Disease Epidemiology, London School of Hygiene & Tropical Medicine, London, WC1E 7HT UK; 4 Department of Biology, Pennsylvania State University, State College, 16801 USA; 5 Bill & Melinda Gates Foundation, Seattle, 98109 USA

**Keywords:** Health impact modeling, Measles, Vaccination, Time-dependent risk

## Abstract

**Background:**

Dynamic modeling is commonly used to evaluate direct and indirect effects of interventions on infectious disease incidence. The risk of secondary outcomes (e.g., death) attributable to infection may depend on the underlying disease incidence targeted by the intervention. Consequently, the impact of interventions (e.g., the difference in vaccination and no-vaccination scenarios) on secondary outcomes may not be proportional to the reduction in disease incidence. Here, we illustrate the estimation of the impact of vaccination on measles mortality, where case fatality ratios (CFRs) are a function of dynamically changing measles incidence.

**Methods:**

We used a previously published model of measles CFR that depends on incidence and vaccine coverage to illustrate the effects of (1) assuming higher CFR in “no-vaccination” scenarios, (2) time-varying CFRs over the past, and (3) time-varying CFRs in future projections on measles impact estimation. We used modeled CFRs in alternative scenarios to estimate measles deaths from 2000 to 2030 in 112 low- and middle-income countries using two models of measles transmission: Pennsylvania State University (PSU) and DynaMICE. We evaluated how different assumptions on future vaccine coverage, measles incidence, and CFR levels in “no-vaccination” scenarios affect the estimation of future deaths averted by measles vaccination.

**Results:**

Across 2000–2030, when CFRs are separately estimated for the “no-vaccination” scenario, the measles deaths averted estimated by PSU increased from 85.8% with constant CFRs to 86.8% with CFRs varying 2000–2018 and then held constant or 85.9% with CFRs varying across the entire time period and by DynaMICE changed from 92.0 to 92.4% or 91.9% in the same scenarios, respectively. By aligning both the “vaccination” and “no-vaccination” scenarios with time-variant measles CFR estimates, as opposed to assuming constant CFRs, the number of deaths averted in the vaccination scenarios was larger in historical years and lower in future years.

**Conclusions:**

To assess the consequences of health interventions, impact estimates should consider the effect of “no-intervention” scenario assumptions on model parameters, such as measles CFR, in order to project estimated impact for alternative scenarios according to intervention strategies and investment decisions.

## Background

Model-based estimation has been widely used to evaluate the impact of infectious disease intervention programs outside of empirical observations [1]. There are myriad policy interests in both retrospective program evaluation to estimate the effect of a previously implemented program and prospective program evaluation to project the impact of different intervention options in the future. In both types of program evaluation, the program impact is often quantified by comparing the estimated effects in the scenario with the intervention against those in a scenario without the intervention (e.g., with and without vaccination). Under this framework, detailed methodological considerations defining both the “intervention” and “no-intervention” scenarios are a necessary condition to minimize bias in program effect estimates.

The most common approach in developing the “no-intervention” scenario is to “switch off” the program in the model. In regression models, this “switch off” can be incorporated by including an indicator variable for program implementation. In mechanistic simulation models, it can be modeled by setting the uptake of the intervention to zero in scenarios without program implementation while keeping all other parameters consistent with scenario(s) where the program is implemented. For both types of models, we can evaluate the intervention impact as the difference in the outcome of interest, such as the number of deaths under the intervention and no-intervention scenarios. Many health impact models assume risks of infectious disease health outcomes conditional on infection, such as case fatality ratios (CFRs), are independent of disease incidence and health system characteristics. However, there is evidence that the outcome of infection and consequences of infection can depend on the health system burden [2–8].

An example of such an intervention is measles vaccination. Due to limited primary data of measles CFR, for many years, the impact of measles vaccine has been estimated by simulation models that assume constant measles CFR over time [9, 10]. However, a recently updated meta-analysis [5] showed a decreasing trend in measles CFR in low- and middle-income countries (LMICs) and found that this is associated with trends in measles vaccination coverage [11], measles incidence [5], and under-five mortality [12]. In most settings, these factors have varied over time and may continue to change in the future, either continuing their past trends or potentially reversing direction following the COVID-19 pandemic [13, 14].

Consequently, in modeling studies that aim to evaluate the impact of a measles vaccination program, changes in the health system are an important aspect to consider as it is related to the risk of disease outcome. As disease management and health system capacity improve over time, we would expect risks of disease outcome to improve over time. Likewise, changes to the population-level risks of disease outcome could be negatively impacted by changes to preventive measures such as vaccination coverage. These observations motivate the design of this study to evaluate the consequences of more realistic assumptions that affect model predictions in the “vaccination” and “no-vaccination” scenarios, accounting for time-dependent elements.

Using measles as a case study, we propose a methodological innovation to model CFR dynamically, which addresses both: (1) dependence of CFR on incidence and other health system characteristics and (2) calculating impact with due consideration for the “no-vaccination” scenario. This paper is organized into two parts. In part I, we used a log-linear regression model to evaluate three different sets of scenarios where covariates used to estimate measles CFR can be time-variant or time-invariant. As a baseline, we used the current practice of assuming time- and strategy-invariant CFRs as described in Wolfson et al. [10]. We also considered two other scenarios in which CFRs depend on covariates that change over time, as informed by our previous study where we showed that CFRs have changed over time from 1980 to 2014 [5]. First, we only allowed for CFRs to change over past time according to the log-linear model, but hold CFRs constant at 2018 levels between 2019 and 2030. This reflects our uncertainty in whether past changes in CFRs will continue into the future. Second, we allowed CFRs to change according to the log-linear model between 2000 and 2030. In part II of this paper, we established an array of “no-vaccination” scenarios of measles vaccination programs in low- and middle-income countries (LMICs), with different assumptions about the trend of CFR estimates obtained from part I.

## Results

The scenario 0 CFRs were stratified by age (</≥ 5 years) for each country; on average across all LMICs, these CFRs were 2.1% for children less than 5 years of age and 1.0% for children 5 years of age and older (scenarios defined in Table 1). In comparison, the estimated time-varying CFRs ranged from 3.7% (2.3–6.3%) in the year 2000 to 1.0% (0.4–3.1%) in the year 2030 for children under five on average across all LMICs, and 1.2% (0.4–3.7%) in 2000 to 0.3% (0.1–1.4%) in 2030 for children 5 years of age and older. In the year 2018, these estimates were 1.6% (0.7–3.7%) for children under five and 0.5% (0.1–1.9%) for children five and older. The estimated CFRs from 2000 to 2030 by under-five mortality rate and region are listed in Appendix 1.

**Table 1:** Analytic scenarios

Scenario	Model	Time-varying period	Constant period	No-vaccination scenario
Scenario 0	Constant CFRs [9, 10]	NA	2000–2030	(a) Constant CFRs
Scenario 1	Time-varying CFRs [5]	2000–2018	2019–2030	(a) Constant CFRs
				(b) Time-varying CFRs
Scenario 2	Time-varying CFRs [5]	2000–2030	NA	(a) Constant CFRs
				(b) Time-varying CFRs

Note: *CFR* case fatality ratio, *NA* not applicable

### Impact of constant CFRs in “no-vaccination” scenario

**Fig. 1:**
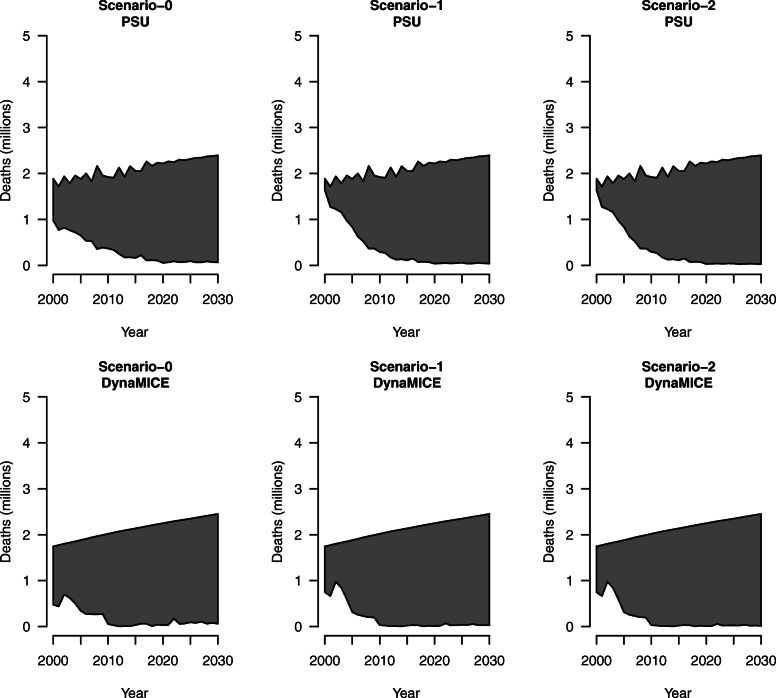
Measles deaths by analytic scenario for 112 countries across 2000 to 2030, assuming a constant case fatality ratio in “no-vaccination” scenario for Pennsylvania State University (PSU) model and DynaMICE model. Note: The top line of each shaded area shows estimated measles deaths in the “no-vaccination” scenario, and the bottom line shows estimated measles deaths in the “vaccination” scenario. The shaded region represents the amount of measles deaths averted by vaccination

Scenario 1 and scenario 2, which assumed time-varying CFRs in the vaccination scenario and constant CFRs in the no-vaccination scenario, resulted in fewer deaths averted than scenario 0, which assumed constant CFRs in both the vaccination and no-vaccination scenarios, for 112 LMICs; this pattern is consistent for projections from both the PSU and DynaMICE models (Table 2). The percent reductions in measles deaths due to vaccination between years 2000 and 2030 under scenario 1 (83.3%; 95% uncertainty range: 70.8–89.9%) and scenario 2 (83.5%; 71.1–90.1%) were both less than the percent reduction in measles deaths under scenario 0 (85.8%) in the PSU model, and correspondingly estimates for scenario 1 (91.6%; 84.4–95.1%) and scenario 2 (91.7%; 84.6–95.2%) were less than scenario 0 (92.0%) in the DynaMICE model. Figure 1 displays these results graphically: the top line of each shaded area shows estimated measles deaths in the “no-vaccination” scenario and the bottom line shows estimated measles deaths in the “vaccination” scenario. The shaded region represents the amount of measles deaths averted by vaccination. In these plots, the “no-vaccination” upper bound of estimated measles deaths for each model remains the same across each analytic scenario.

**Table 2:** Measles deaths averted due to vaccination for 112 countries across 2000 to 2030, assuming a constant case fatality ratio (CFR) in “no-vaccination” scenario and percent reduction compared to no vaccination

Model	Scenario	Time-varying period	Deaths averted (millions) 2000–2018	Deaths averted (millions) 2019–2030	Deaths averted (millions) 2000–2030
PSU	Scenario 0	NA	29.3	26.8	56.1
			77.7%	96.8%	85.8%
	Scenario 1	2000–2018	27.3 (19.9–31.4)	27.1 (26.4–27.4)	54.4 (46.3–58.8)
			72.5% (52.8–83.2%)	98.0% (95.3–99.1%)	83.3% (70.8–89.9%)
	Scenario 2	2000–2030	27.3 (19.9–31.4)	27.3 (26.6–27.5)	54.6 (46.5–58.9)
			72.5% (52.8–83.2%)	98.5% (95.9–99.4%)	83.5% (71.1–90.1%)
DynaMICE	Scenario 0	NA	33.3	27.2	60.5
			88.3%	96.9%	92.0%
	Scenario 1	2000–2018	32.5 (28.5–34.6)	27.7 (27.1–27.9)	60.2 (55.5–62.6)
			86.3% (75.5–91.9%)	98.7% (96.4–99.5%)	91.6% (84.4–95.1%)
	Scenario 2	2000–2030	32.5 (28.5–34.6)	27.8 (27.2–28.0)	60.3 (55.7–62.6)
			86.3% (75.5–91.9%)	99.0% (97.0–99.7%)	91.7% (84.6–95.2%)

Note: The first line for each scenario presents measles deaths averted due to measles vaccination compared to no vaccination for 112 countries aggregated across 2000 to 2030 in millions. The second line for each scenario presents the associated percent reduction in measles deaths compared to no vaccination. The 95% uncertainty intervals across 1000 draws of CFR model parameters are included in parentheses for both measles deaths averted and percent reductions. *PSU* Pennsylvania State University model, *DynaMICE* DynaMICE model developed at the London School of Hygiene & Tropical Medicine

### Impact of matching CFR estimation approach in “no-vaccination” scenario

**Fig. 2:**
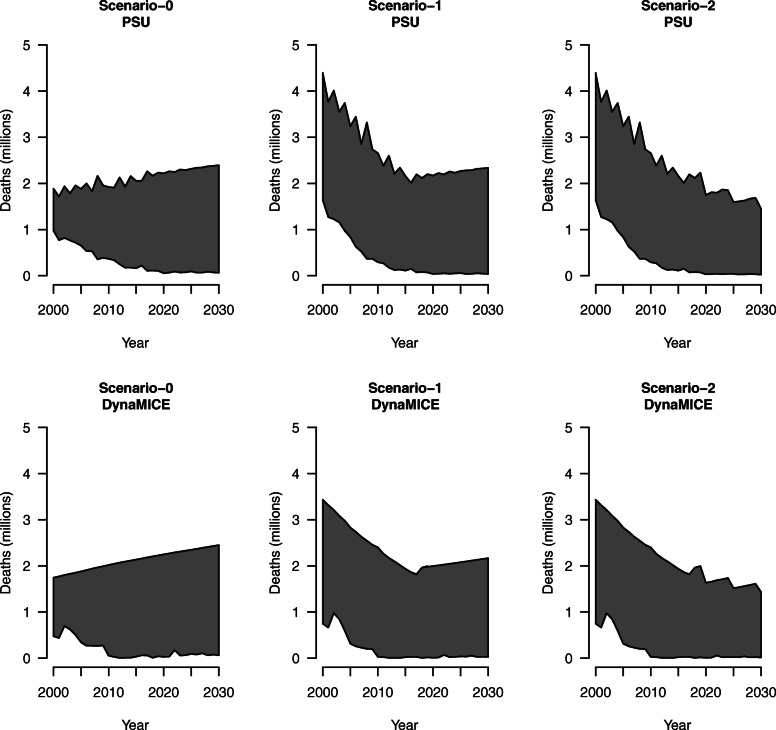
Measles deaths by analytic scenario for 112 countries across 2000 to 2030, assuming a time-varying case fatality ratio in “no-vaccination” scenario for Pennsylvania State University (PSU) model and DynaMICE model. Note: The top line of each shaded area shows estimated measles deaths in the “no-vaccination” scenario, and the bottom line shows estimated measles deaths in the “vaccination” scenario. The shaded region represents the amount of measles deaths averted by vaccination

Scenario 0 assumed constant CFRs under both the “vaccination” scenario and the “no-vaccination” scenario. Scenario 1 and scenario 2 assumed estimated CFRs according to the time-varying, incidence-based methodology for both the “vaccination” scenario and the “no-vaccination” scenario. The percent reduction in measles deaths between 2000 and 2030 under scenario 1 (86.8%; 79.2–91.2%) and scenario 2 (85.9%; 77.4–90.9%) was both greater than the percent reduction in measles deaths under scenario 0 (85.8%) in the PSU model, and estimates for scenario 1 (92.4%; 89.5–94.4%) were more than scenario 0 (92.0%) which is more than scenario 2 (91.9%; 88.7–94.3%) in the DynaMICE model (Table 3). Figure 2 displays these results graphically. In these plots, the bottom line representing the “vaccination” scenario remains the same as in Fig. 1, but the top line representing the “no-vaccination” scenario has now changed to reflect time-varying measles CFR estimation.

**Table 3:** Measles deaths averted due to vaccination for 112 countries across 2000 to 2030, assuming a time-varying case fatality ratio (CFR) in “no-vaccination” scenario and percent reduction compared to no vaccination

Model	Scenario	Time-varying period	Deaths averted (millions) 2000–2018	Deaths averted (millions) 2019–2030	Deaths averted (millions) 2000–2030
PSU	Scenario 0	NA	29.3	26.8	56.1
			77.7%	96.8%	85.8%
	Scenario 1	2000–2018	45.4 (16.9–114.9)	26.5 (8.1–81.8)	71.9 (25.1–196.8)
			81.4% (72.8–86.6%)	97.9% (97.1–98.4%)	86.8% (79.2–91.2%)
	Scenario 2	2000–2030	45.4 (16.9–114.9)	20.5 (5.3–71.4)	65.9 (22.2–189.1)
			81.4% (72.8–86.6%)	98.0% (96.9–98.5%)	85.9% (77.4–90.9%)
DynaMICE	Scenario 0	NA	33.3	27.2	60.5
			88.3%	96.9%	92.0%
	Scenario 1	2000–2018	42.6 (17.7–103.8)	24.5 (9.5–70.0)	67.1 (27.3–173.9)
			89.2% (85.3–91.8%)	98.5% (98.5–98.6%)	92.4% (89.5–94.4%)
	Scenario 2	2000–2030	42.6 (17.7–103.8)	19.4 (7.1–63.0)	62.0 (24.8–166.9)
			89.2% (85.3–91.8%)	98.6% (98.6–98.7%)	91.9% (88.7–94.3%)

Note: The first line for each scenario presents measles deaths averted due to measles vaccination compared to no vaccination for 112 countries aggregated across 2000 to 2030 in millions. The second line for each scenario presents the associated percent reduction in measles deaths compared to no vaccination. The 95% uncertainty intervals across 1000 draws of CFR model parameters are included in parentheses for both measles deaths averted and percent reductions. *PSU* Pennsylvania State University model, *DynaMICE* DynaMICE model developed at the London School of Hygiene & Tropical Medicine

## Discussion

In this study, we illustrated the effect of important considerations for estimating measles CFRs in the evaluation of measles vaccination impact through alternative “vaccination” and “no-vaccination” scenarios. Our aim was to provide evidence that when measles CFR is dependent on factors such as the incidence of measles and the presence of vaccination, the impact of the vaccine program on mortality risk would depend on these contexts as well. In order to reflect this context dependence, estimates of measles CFR should be dynamic in time and reactive to differences in scenarios with a transparent methodology that can produce reproducible estimates. Assuming constant measles CFRs produces impact (number of measles cases averted) that grows over time (due to the growth in cases as population grows), but may overestimate the number of deaths that can be averted by measles vaccination in prospective program evaluation and underestimate deaths averted by measles vaccination historically and devalue past gains in retrospective program evaluation. On the other hand, assuming CFRs that decline in the future, in a way that is consistent with empirical observation [5], leads to an impact that is decreasing in the future (because CFRs decline faster than population growth). Recognizing this trend may provide an opportunity to capitalize on these improvements in order to accelerate that decline and create a world in which no (or very few) children die of measles.

The underlying model for measles CFR estimation assumes a declining CFR trend over time, consistent with previous studies [5]. This likely captures the effects of covariates such as nutrition and health care access that are not explicitly included in the CFR model. However, it is unclear whether measles CFRs will continue to decrease into the future, particularly given disruptions to vaccination coverage and access to routine health services due to the COVID-19 pandemic. However, the time-variant, context-dependent approach to estimating measles CFRs can take new covariate data into consideration in subsequent analyses. In this study, we included a scenario to fix future projections of measles CFRs at 2018 levels as an alternative scenario assuming no additional changes to measles CFR from the current context. Specifically, by estimating measles mortality with time-variant CFRs from 2000 to 2018 rather than constant CFRs for the vaccination scenario, the percent reduction in measles deaths due to vaccination decreased from 77.7 to 72.5% (52.8–83.2%) and 88.3 to 86.3% (75.5–91.9%) in the PSU and DynaMICE models, respectively. This reflects higher vaccine coverage in the later part of this time period. However, by aligning both the “vaccination” and “no-vaccination” scenarios with time-variant measles CFR estimates from 2000 to 2018, the percent reduction in measles deaths increased from 72.5 to 81.4% (72.8–86.6%) and 86.3 to 89.2% (85.3–91.8%) in the PSU and DynaMICE models, respectively.

Despite different approaches, the PSU and DynaMICE models produced similar results, as shown in Figs. 1 and 2 displaying total measles deaths over time with the same overall magnitude and trend in both models. There are larger differences (in relative terms) in countries with high reported coverage (either high routine coverage or frequent high-coverage supplementary immunization activities) but high measles burden, as the DynaMICE model is more optimistic in these settings than the PSU model due to differences in the way the two models convert population immunity levels to estimated measles incidence [15–18]. However, these differences do not affect the estimates aggregated across 112 LMICs, as these countries contribute much less to the overall global burden. The visualizations presented in Figs. 1 and 2 highlight that the differences in mortality by year with the dynamic process showed much greater mortality in historical years and lower mortality in future years compared to constant CFRs. In addition, these reductions reflect measles vaccination impact across 112 LMICs, representing a difference of 10–18 million deaths averted. When matching the CFR estimation approach between the “no-vaccination” and “vaccination” scenarios in Fig. 2, the rebound effect in estimated measles deaths seen in scenario 1 holding CFRs constant beyond 2018 is due to increasing population growth, despite the constant CFRs. Additionally, the tighter bounds seen in Table 3 are due to estimating CFRs separately for the “vaccination” and “no-vaccination” scenarios, compared to assuming constant CFRs for the “no-vaccination” scenario in Table 2.

There are several common approaches in the literature that account for the uncertainty in CFRs in vaccination program evaluation. To name a few, in a prospective program evaluation study where Nonvignon et al. assessed the impact of rotavirus vaccination in Ghana from 2012 to 2031, the authors used a one-way sensitivity analysis to account for their uncertainty in CFR on their model estimate [19]. In another prospective program evaluation study of pneumococcal conjugate vaccine (PCV13) in India, Krishnamoorthy et al. conducted scenario analyses in which they varied parameter values of mortality, disease event rates, vaccine efficacy, coverage projections, and costs by 10% to evaluate the impact of the program in the most and least favorable scenarios [20]. In a study evaluating the impact of past influenza vaccination in the Netherlands, Backer et al. conducted a probabilistic sensitivity analysis to assess how the uncertainty of their model parameters affected their effect estimates, where CFR was modeled as a normal distribution [21]. Methodological approaches such as these capture the uncertainty in the parameter values of CFR in the model, but they do not automatically account for the potential time dependence of CFR. To account for the time trend in CFRs, health impact models can rely on global burden estimates of disease-specific deaths to derive proportional mortality [22] and statistical models like autoregressive integrated moving average models (ARIMA) have also been used in the vaccine literature [23]. However, in methods that make one-step-ahead forecasts such as ARIMA models, long-term forecasting may not be reliable.

In contrast, our study demonstrated a recursive approach where we leveraged a regression model and transmission dynamic models to account for the time dependence of CFRs in the estimation of vaccine impact on mortality. Additionally, by using the incidence estimates obtained from a transmission dynamic model as a covariate in our regression model as well as scenario-specific estimates of CFR values in this transmission dynamic model to forecast the impact of vaccination, we were able to capture the long-term dynamic between CFRs, vaccine coverage, and measles incidence in our estimation of vaccine impact on mortality. Furthermore, by explicitly considering the time trend in CFRs in “vaccination” and “no-vaccination” scenarios, we demonstrated that the assumptions made in “no-vaccination” scenarios can affect the estimation of the public health impact of vaccines and other prevention policies on mortality.

There are several limitations to this analysis. First, the functional form for the relationship between the analyzed covariates and measles CFRs was informed by a limited data set of varying quality, as described previously [5]. The Immunization and Vaccine-related Implementation Research Advisory Committee (IVIR-AC) of the WHO in its recent recommendations noted the need for ongoing primary data collection, including “investments in strengthening outbreak investigation and evaluation activities to generate additional primary data” and the “creation of a standard CFR study protocol and a structured data collection tool to improve comparability of studies” [24]. Second, the log-linear model to predict measles CFR does not necessarily represent causal relationships between the covariates of interest and the outcome. Additional factors that are important considerations for mortality risk, such as malnutrition and treatment status of the individual cases, would require future data collection efforts to supplement currently available data on measles CFRs [5]. We addressed these limitations by estimating two different versions of the “no-vaccination” scenario. While the causal effect of variables not included in the model might be captured in the variables we included, it is unclear which variables should apply to the “no-vaccination” case. Third, there may be additional uncertainty in the impact estimates according to the assumptions of each measles transmission model, described previously [9, 15, 25, 26].

## Conclusions

In order to better estimate the impact of public health interventions, this study can shed light on the effects of alternative assumptions to project future scenarios evaluating intervention impact and provide guidance for developing appropriate “no-vaccination” scenarios. We would expect to see similar relationships between disease management and health system capacity with the risks of disease outcome over time. For example, hospital-fatality risks for COVID-19 tend to increase as the availability of hospital beds and ventilators decreases; on the other hand, there are temporal changes in fatality risk due to improvements in treatment. To assess the consequences of public health interventions, impact estimates should consider the effect of “no-intervention” scenario assumptions on health impact model parameters such as measles CFR, in order to serve the goals of both: estimating the historical and current impact of interventions and projecting estimated impact for alternative scenarios according to intervention strategies and investment decisions. As additional and improved empirical evidence of program implementation becomes available to inform predictive models, we can continue to improve predictions and uncertainty of the effect of public health interventions, such as measles vaccination.

## Methods

### Model overview

We used a previously published log-linear projection model relating CFR to measles incidence, time, and other factors [5], as shown in the following equation:

$$ \ln (CFR)={\beta}_0+{\beta}_1\ast Year+{\beta}_2\ast MCV1\  coverage+{\beta}_3\ast Community\ indicator+{\beta}_4\ast Under\ 5\  indicator+{\beta}_5\ast Attack\ rate+{\beta}_6\ast \ln \left(U5 MR\right)+{\beta}_7\ast \ln \left( Population\ density\right)+{\beta}_8\ast \ln (TFR)+{\beta}_9\ast Percentage\ urban, $$

where *MCV*1 *coverage* = estimated coverage of the routine first dose of measles-containing vaccine (MCV1) [11], *Community indicator* = an indicator for community-based rather than hospital-based measles, *Under* 5 *indicator* = an indicator for children under 5 years old, *Attack rate* = an approximation of measles attack rate (estimated measles incidence divided by annual birth cohort) [12, 27], *U*5*MR* = all-cause under-five mortality rate per 1000 live births [12], *Population density* = population density per square kilometer of land area [12], *TFR* = total fertility rate [12], and *Percentage urban* = percentage of the population living in urban areas [12].

We stratified the population in 112 LMICs by (i) under-five mortality rate (</≥ 50 per 1000 live births) and (ii) world region (Global Burden of Disease regions [28]) and presented all stratifications by age (</≥ 5 years). The full list of countries by these stratifications is included in Appendix 2. For each of these strata, we estimated the measles CFRs across 2000–2030.

The log-linear model was fit to a set of measles CFRs from studies published between 1980 and 2016 [5]. To address uncertainty in our CFR estimations, we quantified the asymptotic variance of the estimated CFRs for each stratum by drawing from a multivariate normal distribution (*n* = 1000), with the means equal to the maximum likelihood estimates (MLE) of the coefficients and the variance–covariance matrix being the variance–covariance of the MLE estimates, all extracted from the regression outputs. The covariates included 2019 World Bank development indicators [12] and estimates of MCV1 coverage between 2000 and 2018 [11], which allowed us to extend the capability of the model to estimate CFRs up to 2018. The measles incidence estimates required for the model formula were generated from two separate, published measles transmission models used by the Vaccine Impact Modelling Consortium (VIMC) to generate global measles vaccine impact estimates for 2000–2018: the Pennsylvania State University (PSU) model and the DynaMICE model developed at the London School of Hygiene & Tropical Medicine (described in Appendices 3 and 4) [29]. The two measles models estimated measles incidence in the case of both “vaccination” and “no-vaccination” scenarios.

The log-linear model was subsequently used to estimate future CFRs from 2019 to 2030 in “vaccination” and “no-vaccination” scenarios in the second part of our analyses. We used projected data for covariates, including under-five mortality rate, total fertility rate, percentage of population in urban areas, and population density [30]. Population density was available with annual projections, whereas under-five mortality, total fertility rate, and urban percentage were available by 5-year increments [30]. Future MCV1 coverage was projected by the authors from 2018 World Health Organization (WHO) and UNICEF estimates of national immunization coverage (WUENIC) [11], assuming a 1% coverage increase per year in line with prior analyses [31]. Future MCV1 coverage was capped at 95%, unless a country had a higher projected coverage as of 2018 in which case the coverage was capped at the maximum coverage reached. In the “no-vaccination” scenario, MCV1 coverage was assumed to be zero.

### Part I: CFR scenarios assuming vaccination

We estimated measles CFRs in three scenarios (Table 1). In scenario 0, we relied on previous estimates of measles CFRs [9], based on a descriptive analysis [10], assumed to be constant across 2000 to 2030. In scenario 1, we estimated CFRs from 2000 to 2018 using the log-linear model described above with covariates from 2000 to 2018. This allowed us to examine how these time-variant covariates would affect CFR estimates over time, under the assumption that there is a correlation between CFR, vaccination coverage, measles incidence, and under-five mortality as outlined in the previous analysis [5], which resulted in a declining trend of measles CFR. In this scenario, the 2018 CFR estimate was assumed to be constant from 2018 to 2030 (held at 2018 levels predicted by the log-linear model), given the uncertainty in the trend of covariates due to unknown future data.

In scenario 2, we extended the CFR projection end year from 2018 to 2030, using projected data for covariates, described above. The incidence estimates from 2019 to 2030 were generated from the same measles transmission models used in scenario 1.

Subsequently, we used the CFR estimates to quantify the impact of estimated CFRs by analytic scenario on estimates of measles deaths from 2000 to 2030, described in part II, using the PSU and DynaMICE models of measles transmission [9, 25]. Measles deaths averted by vaccination were calculated in comparison with a strategy of no measles vaccination in 112 LMICs. We used R statistical software, version 3.6.1, for all analyses [32].

### Part II: CFR scenarios assuming “no-vaccination”

In order to calculate the impact of vaccination, we need to project the burden of disease in the absence of vaccination. Because we had no empirical support for what CFR would be in the absence of vaccination, we tested two alternative approaches. Specifically, we compared the analytic scenarios 1 and 2 in Table 1 to two alternative “no-vaccination” scenarios that assumed (a) CFRs remain constant and (b) time-varying CFRs according to the approach used in the comparator “vaccination” scenario. We evaluated how the different assumptions in the “no-vaccination” scenarios affected our estimation on future deaths averted by measles vaccination. These impact estimates were compared to impact estimates where age-specific CFRs in the “no-vaccination scenario” were assumed to be the same as in the corresponding “vaccination” scenario.

## Data Availability

The datasets generated during and/or analyzed during the current study are available from the corresponding author on reasonable request.

## Appendix 1: Estimated measles case fatality ratios (CFRs) from 2000 to 2030 by model, vaccination scenario, age, under-five mortality rate (U5MR), and region

*Under-five CFRs under vaccination (PSU)*

1. U5MR < 50
2. U5MR ≥ 50

*Over-five CFRs under vaccination (PSU)*

1. U5MR < 50
2. U5MR ≥ 50

*Under-five CFRs under no vaccination (PSU)*

1. U5MR < 50
2. U5MR ≥ 50

*Over-five CFRs under no vaccination (PSU)*

1. U5MR < 50
2. U5MR ≥ 50

*Under-five CFRs under vaccination (DynaMICE)*

1. U5MR < 50
2. U5MR ≥ 50

Note: The LAC region at ≥50 U5MR only includes the country of Haiti, which experienced a significant shock to covariates due to the 2010 earthquake.

*Over-five CFRs under vaccination (DynaMICE)*

1. U5MR < 50
2. U5MR ≥ 50

Note: The LAC region at ≥ 50 U5MR only includes the country of Haiti, which experienced a significant shock to covariates due to the 2010 earthquake.

*Under-five CFRs under no vaccination (DynaMICE)*

1. U5MR < 50
2. U5MR ≥ 50

Note: The LAC region at ≥ 50 U5MR only includes the country of Haiti, which experienced a significant shock to covariates due to the 2010 earthquake.

*Over-five CFRs under no vaccination (DynaMICE)*

1. U5MR < 50
2. U5MR ≥ 50

Note: The LAC region at ≥ 50 U5MR only includes the country of Haiti, which experienced a significant shock to covariates due to the 2010 earthquake.

Note: *EURA*, Central Europe, Eastern Europe, and Central Asia; *LAC*, Latin America and the Caribbean; *MENA*, North Africa and Middle East; *SA*, South Asia; *SEAO*, South-East Asia, East Asia, and Oceania; *SSA*, sub-Saharan Africa; *< 50*, less than 50 deaths per 1000 live births; *≥ 50*, greater than or equal to 50 deaths per 1000 live births.

## Appendix 2: Country list by under-five mortality rate (U5MR) and region

**Table:**

Country	Region	U5MR
Afghanistan	MENA	≥ 50
Albania	EURA	< 50
Algeria	MENA	< 50
Angola	SSA	≥ 50
Argentina	LAC	< 50
Armenia	EURA	< 50
Azerbaijan	EURA	< 50
Bangladesh	SA	< 50
Belarus	EURA	< 50
Belize	LAC	< 50
Benin	SSA	≥ 50
Bhutan	SA	< 50
Bolivia	LAC	< 50
Bosnia and Herzegovina	EURA	< 50
Botswana	SSA	< 50
Brazil	LAC	< 50
Bulgaria	EURA	< 50
Burkina Faso	SSA	≥ 50
Burundi	SSA	≥ 50
Cabo Verde	SSA	< 50
Cambodia	SEAO	< 50
Cameroon	SSA	≥50
Central African Republic	SSA	≥ 50
Chad	SSA	≥50
China	SEAO	< 50
Colombia	LAC	< 50
Comoros	SSA	≥50
Congo DR	SSA	≥50
Congo	SSA	< 50
Costa Rica	LAC	< 50
Côte d'Ivoire	SSA	≥ 50
Cuba	LAC	< 50
Djibouti	SSA	≥ 50
Dominica	LAC	< 50
Dominican Republic	LAC	< 50
Ecuador	LAC	< 50
Egypt	MENA	< 50
El Salvador	LAC	< 50
Equatorial Guinea	SSA	≥ 50
Eritrea	SSA	< 50
Ethiopia	SSA	≥50
Fiji	SEAO	< 50
Gabon	SSA	≥ 50
Gambia	SSA	≥ 50
Georgia	EURA	< 50
Ghana	SSA	≥50
Grenada	LAC	< 50
Guatemala	LAC	< 50
Guinea	SSA	≥50
Guinea-Bissau	SSA	≥50
Guyana	LAC	< 50
Haiti	LAC	≥ 50
Honduras	LAC	< 50
India	SA	< 50
Indonesia	SEAO	< 50
Iran	MENA	< 50
Iraq	MENA	< 50
Jamaica	LAC	< 50
Jordan	MENA	< 50
Kazakhstan	EURA	< 50
Kenya	SSA	< 50
Kiribati	SEAO	≥50
Korea DPR	SEAO	< 50
Kyrgyz Republic	EURA	< 50
Lao PDR	SEAO	≥ 50
Lebanon	MENA	< 50
Lesotho	SSA	≥ 50
Liberia	SSA	≥ 50
Libya	MENA	< 50
Macedonia	EURA	< 50
Madagascar	SSA	< 50
Malawi	SSA	≥50
Malaysia	SEAO	< 50
Maldives	SEAO	< 50
Mali	SSA	≥ 50
Marshall Islands	SEAO	< 50
Mauritania	SSA	≥ 50
Mauritius	SEAO	< 50
Mexico	LAC	< 50
Micronesia	SEAO	< 50
Moldova	EURA	< 50
Mongolia	EURA	< 50
Montenegro	EURA	< 50
Morocco	MENA	< 50
Mozambique	SSA	≥ 50
Myanmar	SEAO	≥ 50
Namibia	SSA	< 50
Nepal	SA	< 50
Nicaragua	LAC	< 50
Niger	SSA	≥ 50
Nigeria	SSA	≥ 50
Pakistan	SA	≥ 50
Palau	SEAO	< 50
Panama	LAC	< 50
Papua New Guinea	SEAO	≥ 50
Paraguay	LAC	< 50
Peru	LAC	< 50
Philippines	SEAO	< 50
Romania	EURA	< 50
Russian Federation	EURA	< 50
Rwanda	SSA	< 50
Samoa	SEAO	< 50
São Tomé and Principe	SSA	< 50
Senegal	SSA	< 50
Serbia	EURA	< 50
Sierra Leone	SSA	≥ 50
Solomon Islands	SEAO	< 50
Somalia	SSA	≥ 50
South Africa	SSA	< 50
South Sudan	SSA	≥ 50
Sri Lanka	SEAO	< 50
St Lucia	LAC	< 50
St Vincent and the Grenadines	LAC	< 50
Sudan	MENA	≥ 50
Suriname	LAC	< 50
Swaziland	SSA	≥ 50
Syrian Arab Republic	MENA	< 50
Tajikistan	EURA	< 50
Tanzania	SSA	< 50
Thailand	SEAO	< 50
Timor-Leste	SEAO	≥ 50
Togo	SSA	≥ 50
Tonga	SEAO	< 50
Tunisia	MENA	< 50
Turkey	MENA	< 50
Turkmenistan	EURA	≥ 50
Tuvalu	SEAO	< 50
Uganda	SSA	≥ 50
Ukraine	EURA	< 50
Uzbekistan	EURA	< 50
Vanuatu	SEAO	< 50
Venezuela	LAC	< 50
Vietnam	SEAO	< 50
Yemen	MENA	< 50
Zambia	SSA	≥ 50
Zimbabwe	SSA	≥ 50

Note: *EURA* Central Europe, Eastern Europe, and Central Asia, *LAC* Latin America and the Caribbean, *MENA* North Africa and Middle East, *SA* South Asia, *SEAO* South-East Asia, East Asia, and Oceania, *SSA* sub-Saharan Africa, *< 50* less than 50 deaths per 1000 live births, *≥ 50* greater than or equal to 50 deaths per 1000 live births

## Appendix 3: DynaMICE model overview^1^

The Dynamic Measles Immunization Calculation Engine (DynaMICE) is a dynamic age-stratified population model of measles transmission dynamics to estimate the public health impact and cost-effectiveness of routine vaccination programs and supplementary immunization activities (SIAs) in low- and middle-income countries.^2^ It was originally developed for work with the World Health Organization and received inputs from investigators at Harvard University and Montreal University as well as LSHTM. It was subsequently used to inform vaccine impact projections for Gavi, the Vaccine Alliance, and the Bill & Melinda Gates Foundation. The model provides a flexible framework that can be adapted to different countries with the aim to study several vaccination scenarios based on available data sources. For example, the model has been adapted to characterize measles transmission and dynamics in India, based on measles data from the Million Deaths Study,^3^ as well as to quantify the impact of adding interventions to measles SIAs in India by interfacing with the Lives Saved Tool (LiST).^4^

As measles is a highly transmissible childhood infection, disease dynamics are inextricably linked to population structure and demographic parameters. To enable precision in the estimation of disease burden and the contact processes that drive transmission, the model is age-stratified to include weekly age groups from birth to 3 years of age, and yearly age groups from 3 to 100 years of age. The underlying epidemic model is a compartmental SIR model, where individuals can either be susceptible (S) to measles, infected (I), or recovered (R) with life-long immunity. After a certain duration of maternal immunity, births replenish the pool of susceptibles that in the absence of vaccination fuel periodic outbreaks of measles driven by the magnitude of the birth rate and the strength of seasonality in transmission parameters. Susceptibles get infected through contact with infected individuals, with mixing determined by age-dependent contact patterns. The contact matrix used for this exercise is the POLYMOD contact matrix for Great Britain^5^ as it was best able to reproduce transmission dynamics across a range of countries, but this can be updated to represent local population age structure. Following infection, individuals either recover and gain life-long immunity or die as described by country-specific age-dependent case fatality ratios (CFRs).

Routine vaccination is modeled through first- and second-dose measles-containing vaccine (MCV1 and MCV2) schedules (corrected for the right cohorts) with the additional option of including SIAs. Vaccines are assumed to be “all or nothing” with effectiveness equal to 84% for the first dose among children under the age of one year, 93% for the first dose among children over the age of 1 year, and 99% for both doses,^6^ with life-long vaccine-induced immunity.

## Appendix 4: PSU model overview^7^

The PSU measles model is a semi-mechanistic, age-structured, discrete time-step, annual SIR model. Unlike conventional SIR models, which describe dynamics at the scale of an infectious generation (TSIR REF)^8^ or finer (basic REF)^9^, it models the aggregate number of cases over 1-year time steps. While this is coarse relative to the time scale of measles transmission, it matches the annual reporting of measles cases available for all countries since approximately 1980 for all countries through the WHO Joint Reporting Form (JRF). To account for the fine-scale dynamics that are being summed over a full year, the model describes the number of infections (*I*_*i*,*t*_) in country *i* and year *t*, and age class *a* as an increasing function of the fraction, *p*_*i*,*t*_ , of the population susceptible in age class *a* at the start of year *t*, *S*_*i*,*t*_:

$$ E\left[{I}_{a,i,t}\right]={p}_{i,t}\ast {S}_{a,i,t}, $$

where *E*[∙] indicates the expectation and *p*_*i*, *t*_ is a country- and year-specific annualized attack rate modeled as:

$$ {p}_{i,t}= invlogit\left(-{\beta}_{0,i}+{\beta}_{1,i}\ast \frac{\sum_a{S}_{i,t}}{N_{i,t}}+{e}_t\right), $$

where invlogit() indicates the inverse logit function, *N*_*i*,*t*_ is the total population size in country *i* and year *t* over all age classes, and *e*_*t*_ is a Gaussian random variable with mean 0 and variance *σ*^2^. The parameters *β*_0, *i*_ , *β*_1, *i*_, and *σ*^2^ are fit to each country independently using a state-space model fitted to observed annual cases reported through the JRF from 1980 to 2016 as described by Eilertson et al.^10^ Historical population and vaccination coverage values are provided by WHO as described by Simons et al.^11^

The number of susceptible individuals in each single-year age class *a* (*a* = 2,…, 25) is equal to the number not infected in the previous year, nor immunized through supplemental immunization activities (SIAs). The number susceptible is further deprecated by the crude death rate. The efficacy of doses administered through SIAs is assumed to be 99%. The number of susceptible individuals in age class *a* = 1 is assumed to be 50% of the annual live birth cohort; this assumes that all children have protective maternal immunity until 6 months of age. Age class *a* = 2 and *a* = m is assumed to receive a first and second dose (respectively) of routine measles vaccination before the start of the time step; thus, the number susceptible is further reduced by the product of the coverage and the efficacy. Efficacy is assumed to be 85% and 93% for the first dose in countries delivering at 9 months and 12 months of age, respectively, and assumed to be 99% for the second dose.

Deaths are calculated by applying an age- and country-specific case fatality ratio (CFR) to each country. CFRs for cases below 59 months of age for all countries were taken from Wolfson et al.^12^; CFR for cases above 59 months of age are assumed to be 50% lower than those applying to under-5 s.

Forward simulations of this model assume random variation in the annual attack rate according to the parameter *σ*^2^. Furthermore, each forward simulation draws *β*_0, *i*_ , *β*_1, *i*_ at random from the joint 95% interval estimate of each parameter. Future vaccination coverage values, for routine and SIAs, are assumed known and future birth and death rates are assumed known.

## Abbreviations

ARIMA: Autoregressive integrated moving average models
CFR: Case fatality ratio
DynaMICE: DynaMICE model developed at the London School of Hygiene & Tropical Medicine
IVIR-AC: Immunization and Vaccine-related Implementation Research Advisory Committee
LMICs: Low- and middle-income countries
MCV1: Routine first dose of measles-containing vaccine
MLE: Maximum likelihood estimates
PSU: Pennsylvania State University
TFR: Total fertility rate
U5MR: All-cause under-five mortality rate per 1000 live births
VIMC: Vaccine Impact Modelling Consortium
WHO: World Health Organization
WUENIC: WHO and UNICEF estimates of national immunization coverage
